# Frailty in People with HIV Is Linked to Inflammation, Bone Health, and T-Cell Exhaustion

**DOI:** 10.1093/infdis/jiag046

**Published:** 2026-02-06

**Authors:** Michael L Freeman, Wendy Fitzgerald, Brian M Clagett, Katelyn A O’Hare, Vikhyath Jonnalagadda, Brigid M Wilson, Leonid Margolis, Kunling Wu, Katherine Tassiopoulos, Carey L Shive, Kristine M Erlandson

**Affiliations:** Rustbelt Center for AIDS Research, Division of Infectious Diseases and HIV Medicine, Department of Medicine, Case Western Reserve University School of Medicine, Cleveland, Ohio, USA; Center for Global Health and Diseases, Department of Pathology, Case Western Reserve University School of Medicine, Cleveland, Ohio, USA; Section on Intercellular Interactions, Eunice Kennedy Shriver National Institute of Child Health and Human Development, National Institutes of Health, Bethesda, Maryland, USA; Rustbelt Center for AIDS Research, Division of Infectious Diseases and HIV Medicine, Department of Medicine, Case Western Reserve University School of Medicine, Cleveland, Ohio, USA; Rustbelt Center for AIDS Research, Division of Infectious Diseases and HIV Medicine, Department of Medicine, Case Western Reserve University School of Medicine, Cleveland, Ohio, USA; Rustbelt Center for AIDS Research, Division of Infectious Diseases and HIV Medicine, Department of Medicine, Case Western Reserve University School of Medicine, Cleveland, Ohio, USA; Geriatric Research, Education, and Clinical Center, Veterans Affairs Northeast Ohio Healthcare System, Cleveland, Ohio, USA; Faculty of Natural Sciences and Medicine, Ilia State University, Tbilisi, Georgia; Center for Biostatistics in AIDS Research, Harvard T.H. Chan School of Public Health, Boston, Massachusetts, USA; Department of Epidemiology, Harvard T.H. Chan School of Public Health, Boston, Massachusetts, USA; Geriatric Research, Education, and Clinical Center, Veterans Affairs Northeast Ohio Healthcare System, Cleveland, Ohio, USA; Department of Pathology, Case Western Reserve University School of Medicine, Cleveland, Ohio, USA; Division of Infectious Diseases, Department of Medicine, University of Colorado Anschutz Medical Campus, Aurora, Colorado, USA

**Keywords:** HIV/AIDS, frailty, inflammation, T cells, senescence-associated secretory phenotype

## Abstract

**Background:**

Little is known about the specific inflammatory networks and immune parameters that drive frailty outcomes in people with HIV (PWH).

**Methods:**

Plasma analytes and T-cell phenotypes from PWH without frailty (0 Fried score, n = 60) and with frailty (≥3 Fried score, n = 60) were measured by Luminex assay or flow cytometry. Multiple least-squares linear regression analysis was used to determine the association of each marker with frailty in unadjusted and adjusted models. Spearman correlations were used to determine the association of plasma analytes with T-cell phenotypes.

**Results:**

We found that 19 of 75 markers measured in plasma were significantly associated with frailty, most of which are downstream of NF-κB signaling and are senescence-associated secretory phenotype (SASP) components. In frail individuals, the proportions of CD4 and CD8 T cells with a naïve phenotype were significantly reduced, and the proportions of CD4 T cells expressing TIGIT and PD-1 were significantly elevated. Of the frailty-associated analytes, we found that only osteoprotegerin and TNF levels were significantly correlated with percent naïve, TIGIT+, and PD-1+ CD4 T cells among PWH with frailty. Osteoprotegerin levels were negatively correlated with CD4/CD8 T-cell ratio.

**Conclusions:**

We found a strong association of the SASP and NF-κB-related inflammation with frailty in PWH. Osteoprotegerin can inhibit osteoclast formation and prevent bone resorption. Low proportion of naïve CD4 T cells and increased TIGIT and PD-1 expression were associated with both osteoprotegerin levels and frailty, suggesting a link between inflammation, T-cell activation, bone health, and frailty in PWH.

## Teaser

We compared plasma inflammatory markers and T-cell phenotypes in people with HIV, with and without frailty. We found that NF-κB-driven senescence-associated secretory phenotype proteins and T-cell exhaustion markers were most related to frailty and to each other.

**(See the Editorial Commentary by Routy and Isnard on pages 975–6.)**

Chronic inflammation associated with aging (known as inflammaging) is a critical driver of morbidities and geriatric syndromes such as frailty, a condition characterized by the accumulation of age-related functional decline and vulnerability to stressors. While advances in antiretroviral therapy (ART) have rendered people with HIV-1 (PWH) with lifespans that are near to those of similarly aged people without HIV (PWoH), elevated plasma levels of inflammatory factors in PWH contribute to increased incidence and earlier onset of non-AIDS morbidities and geriatric syndromes, including frailty [1, 2]. Understanding the specific inflammatory components that drive frailty outcomes will help inform the development of targeted therapeutic interventions that minimize impacts on other pathways resulting in safer, more effective treatments.

The cytokine interleukin (IL)-6 is a central orchestrator of inflammatory pathways and is a major component of the proinflammatory milieu produced by senescent cells, known as the senescence-associated secretory phenotype (SASP) [3]. In PWH, systemic IL-6 levels (1) are significantly upregulated; (2) do not fully normalize with ART; (3) are a strong predictor of non-AIDS comorbidities, including frailty, and of mortality, and (4) are positively correlated with expression of the senescence-associated transcript p16^INK4a^ in T cells [4–7]. In addition, elevated plasma IL-6 levels have been associated with sarcopenia [8, 9], mortality [10], and frailty [11] in elderly PWoH. Furthermore, in a mouse model of inducible IL-6, young mice that were engineered to have elevated systemic IL-6 levels equivalent to levels in old mice developed hallmarks of frailty, including reduced grip strength and disrupted mitochondrial homeostasis in muscle tissues [12]. However, how IL-6 and other inflammatory cytokines are linked to frailty in PWH is unknown.

Chronic inflammation is also a major contributor to alterations in T-cell responses, yet very little is known about the relationships among T cells, inflammation, and frailty. For instance, a recent clinical trial demonstrated that blocking IL-6 signals with the IL-6 receptor (IL-6R) antagonist tocilizumab results in decreased expression of programmed death-1 (PD-1) on CD4 T cells [13], but how PD-1 expression is associated with frailty is unknown, and frailty outcomes were not assessed in the trial. In this report, we compared peripheral blood T-cell phenotypes, including markers of exhaustion (eg, PD-1) and senescence (eg, CD57), and plasma soluble inflammatory markers (including IL-6) in PWH with and without frailty. All participants were enrolled in the HIV Infection, Aging, and Immune Function Long-Term Observational Study (HAILO; A5322), a near decade-long ACTG observational study, with annual frailty assessments [14–16]. By characterizing the immune response and systemic inflammatory milieu in PWH with frailty, our findings may provide novel insight into the mechanisms driving frailty in this highly at-risk population.

## METHODS

### Participants

All participants for this study had been enrolled in ACTG A5322: The HAILO Study, between November 2013 and November 2021 (up to 384 weeks on study), and provided written informed consent in accordance with the Declaration of Helsinki. Frailty was assessed every 48 weeks ± 24 weeks using a modified version of the Fried frailty criteria [17], with frailty defined as the presence of least 3 of the following criteria: unintentional weight loss (shrinking), low physical activity, exhaustion, reduced muscle strength (weakness), and slow walking speed (slowness), as previously described [14–16]. Those with no frailty components were considered robust. Stored plasma and cryopreserved peripheral blood mononuclear cells (PBMCs) were acquired from 60 participants who were robust (Fried score = 0) at entry and remained robust; these participants were matched by age at entry (±5 years), years of follow-up, and sex to 60 donors who were either robust or pre-frail (Fried score = 1 or 2) at entry and became frail. All samples in the frail group were from a time point when the donor exhibited frailty (Fried score ≥ 3). Comorbidities were not considered in the matching process.

### Cytokine Measurement

We used 3 in-house multiplexed bead-based assays to measure 75 cytokines, growth factors, and other proteins (Supplementary Table 1) as described in previous publications [18, 19]. Magnetic beads (Luminex) with distinct spectral signatures (regions) were coupled to protein specific capture antibodies according to the manufacturer's recommendations and stored at 4°C. Plasma samples were thawed and immediately used in assays. Plasma was diluted 1/3 (except for panel 3 in which samples were serially diluted 1/5, 1/125, 1/625, and 1/3125) in ProCartaPlex Platinum Assay Buffer for Plasma Samples (Thermo Fisher) in presence of Heteroblock (150 µg/mL) (Omega Biologicals) to prevent nonspecific interaction between antibodies. Standard curves were run in duplicate in a 1:2 solution of SeraSub (CST Technologies) and plasma diluent. Standards and samples were incubated with bead mixtures overnight at 4°C. Plates were washed 3 times and incubated with mixtures of polyclonal biotinylated anti-protein antibodies (R&D Systems) in assay buffer (1× PBS with 20 mM Tris-HCl, 1% species-specific serum [Gemini Bioproducts] and 0.05% Tween 20) for 1 h at room temperature. Plates were washed 3 times and incubated for 30 minutes with 12 μg/mL streptavidin-phycoerythrin (Thermo Fisher) in PBS. Plates were washed 3 times and beads were resuspended in PBS. Plates were read on a Luminex 200 analyzer with acquisition of a minimum of 100 beads for each region and analyzed using Bioplex Manager software (BioRad). Protein concentrations were determined using 5P regression algorithms. Due to a technical issue, we were unable to acquire plasma analyte data from one PWH with frailty.

### Peripheral Blood Mononuclear Cell and Flow Cytometry

Cryopreserved PBMCs were thawed, washed in complete RPMI media (10% fetal bovine serum, 1% L-glutamine, 1% penicillin/streptomycin), incubated with Live/Dead Aqua viability dye (Thermo Fisher), and stained with antibodies to CD3 (BUV737, clone UCHT1; BD Biosciences), CD4 (BUV395, SK3; BD), CD8 (BV605, SK1; BD), CD45RA (BV650, HI100; BD), CCR7 (PE-CF594, 150503; BD), CD28 (APC-Cy7, CD28.2; BioLegend), CD57 (AF647, HNK-1; BD), PD-1 (BV785, EH12.2H7; BioLegend), TIGIT (BV711, A15153G; BioLegend), CX3CR1 (PerCP-Cy5.5, 2A9-1; BioLegend), TOX (PE, TXRX10; eBioscience), TCF-1 (BV421, S33-966; BD), and CD101 (PE-Cy7, BB27; BioLegend). Following surface antibody labeling, cells were fixed and permeabilized using the True-Nuclear Transcription Factor kit (BioLegend) per manufacturer's instructions, then stained with anti-p16^INK4a^ (FITC, G175-1239; BD). Samples were acquired on an LSR-Fortessa flow cytometer (BD).

### Statistical Analyses

Demographics, HIV viral load, CD4 T-cell count, and chronic conditions were summarized and compared between frail and non-frail groups using χ² and Mann–Whitney tests, where appropriate. For principal component analyses (PCA), the levels of soluble analytes and T-cell phenotypic parameters were converted to z-scores for normalization, then calculated using the prcomp function and visualized with the ggbiplot package running under R (v4.4.2) in RStudio (2025.05.0). The association of frailty with soluble analytes and T-cell phenotypic parameters was determined with 4 models, each analyzed using multiple least-squares linear regression analysis in Prism v10 (GraphPad). Model 1 was univariate. Model 2 was adjusted for age, sex, and race/ethnicity. Model 3 was adjusted for Model 2 parameters and for diabetes mellitus, hypertension, and cardiovascular disease. Model 4 was adjusted for Model 3 parameters and for smoking status. Beta estimates for each parameter were converted to effect size (Cohen's d) for graphing. Plasma analyte data was available for 59 out of 60 participants with frailty; models were performed using donors with available data. Protein–protein interaction visualizations and analyses were generated using the STRING database (v12.0) [20]. Determination of *NFKB1* transcriptional targets was determined using the TRRUST database (v2) [21]. Characterization of SASP status was determined by literature search [22–30]. Correlation analyses were performed using Spearman analysis. Correlogram matrices plotting Spearman correlation coefficient (rho) were generated using the corrplot package, and nodes and edges in the correlation model were plotted using the qgraph package in RStudio [31]. In all cases, differences were considered significant if the *P* value was <.05.

### Study Approval

This study was approved by the Institutional Review Boards at each participating ACTG site and by the Case Western Reserve University Institutional Review Board (#20230181).

## RESULTS

We compared plasma analytes and peripheral blood T-cell phenotypes in the most recent available samples among 120 PWH, 60 of whom were frail (at least 3 Fried frailty criteria) and 60 of whom were robust (0 Fried frailty criteria), matched by age and sex. As shown in Table 1, non-frail and frail PWH were similar by race/ethnicity, body mass index, CD4 T-cell counts, and substance use, but a greater proportion of PWH with frailty were current or former smokers (83% vs 67%, *P* = *.04*) and had hypertension (83% vs 63%, *P* = *.01*), cardiovascular disease (CVD; 27% vs 12%, *P* = *.04*), and diabetes mellitus (30% vs 8%, *P* = *.05*). Fewer PWH with frailty had HIV-1 plasma RNA levels at or below 40 copies/mL (83% vs 97%, *P* = *.02*). While integrase strand transfer inhibitor (INSTI) use may have a protective effect on gait speed [15], switching to an INSTI-based ART regimen may increase the risk of diabetes [32]. Here, INSTI use at the time of sampling was similar between the groups.

**Table 1.** Participant Demographics

	Non-frail	Frail	*P* Value
n	60	60	
Age (y), median (IQR)	58.0 (52.3–64.0)	59.6 (53.3–65.0)	ns
Sex at Birth, n (%)			ns
Male	45 (75)	45 (70)	
Female	15 (25)	15 (25)	
Race/ethnicity, n (%)			ns^a^
Black, non-Hispanic	18 (30.0)	20 (33.3)	
White, non-Hispanic	33 (55.0)	26 (43.3)	
Hispanic, any race	6 (10.0)	13 (21.7)	
Any other race	3 (5.0)	1 (1.7)	
Body mass index, median (IQR)	26.7 (23.1–29.1)	28.3 (23.9–32.1)	ns
CD4 T-cell count (cells/µL), median (IQR)	637 (493–853)	677 (541–829)	ns
CD8 T-cell count (cells/µL), median (IQR)	642 (468–835)	742 (532–1011)	ns
CD4/CD8 ratio	1.01 (0.76–1.45)	0.92 (0.59–1.27)	ns
Plasma HIV RNA ≤ 40 copies/mL, n (%)	58 (96.7)	50 (83.3)	.015
Time on ART (y), median (IQR)	13.6 (9.4–17.4)	13.3 (8.8–17.8)	ns
INSTI use, n (%)	38 (63)	37 (62)	ns
GLP-1 receptor agonist use, n (%)	0 (0)	2 (3.3)	ns
Fried Frailty Score, median (IQR)	0 (0.0–0.0)	3 (3.0–4.0)	na
Fried Frailty Score 3, n (%)	na	40 (66.7)	
Fried Frailty Score 4, n (%)	na	18 (30.0)	
Fried Frailty Score 5, n (%)	na	2 (3.3)	
Substance use			ns^b^
Never, n (%)	18 (30.0)	10 (16.7)	
Prior, n (%)	30 (50.0)	30 (50.0)	
Current, n (%)	12 (20.0)	20 (33.3)	
Smoking			.035^b^
Never, n (%)	20 (33.3)	10 (16.7)	
Prior, n (%)	17 (28.3)	35 (58.3)	
Current, n (%)	23 (38.3)	15 (25.0)	
Hypertension, n (%)	38 (63.3)	50 (83.3)	.013
CVD, n (%)	7 (11.7)	16 (26.7)	.037
Diabetes, n (%)	5 (8.3)	18 (30.0)	.005

Data were presented as median and interquartile range (IQR) or number (%). Significance for continuous variables was determined with Mann–Whitney U test. Significance for contingency analyses was determined with χ² test.

Abbreviations: ns, not significant; na, not applicable; ART, antiretroviral therapy; CVD, cardiovascular disease; INSTI, integrase strand transfer inhibitor; GLP-1, glucagon-like peptide-1.

^a^White, non-Hispanic versus all other categories.

^b^Never versus prior or current.

**Figure 1.**
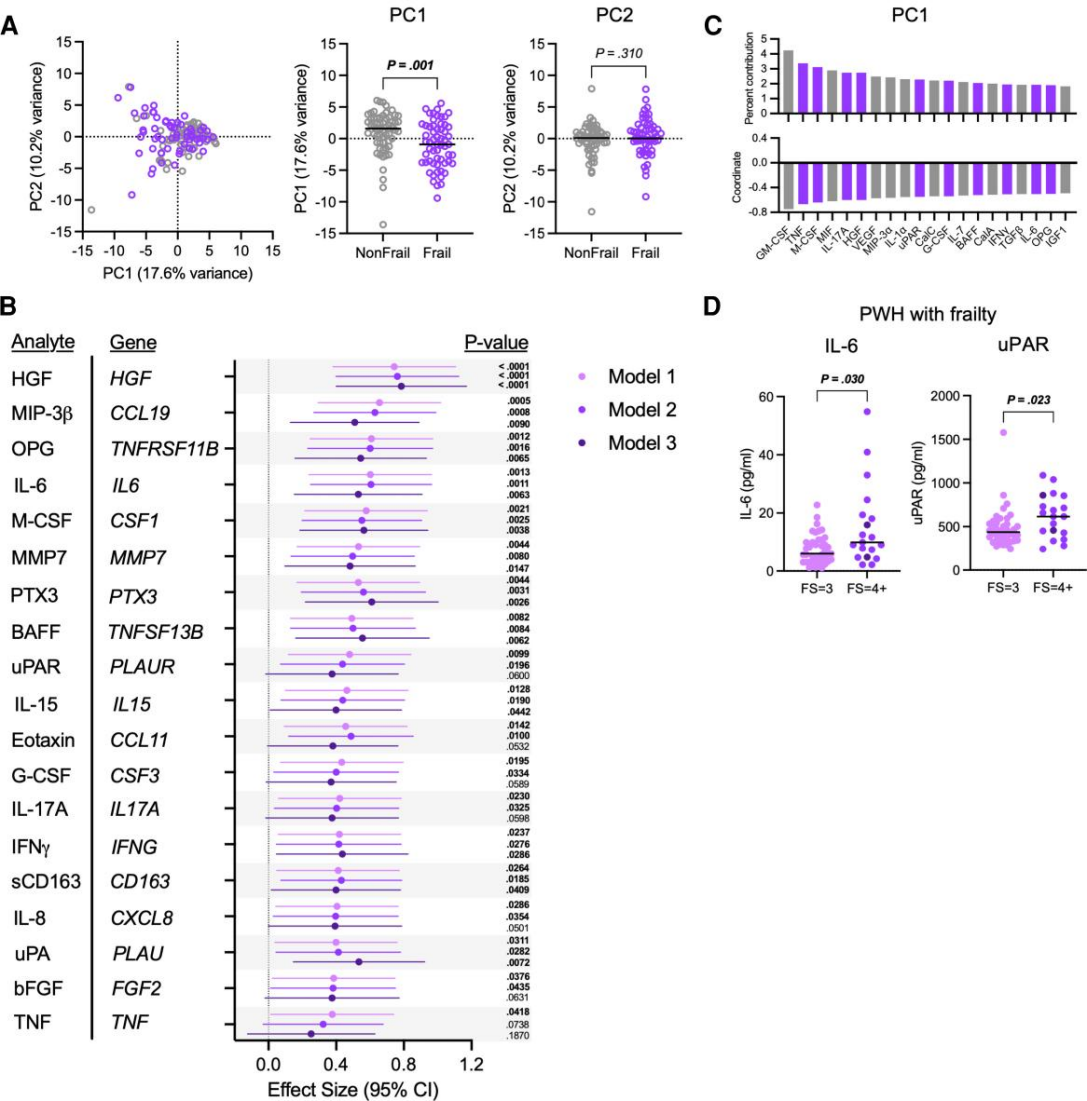
Association of soluble analytes with frailty in people with HIV. Soluble analytes in plasma were measured in people with HIV (PWH) without frailty (non-frail, n = 60) and in PWH with frailty (frail, n = 59). *A*, Left: Principal component analysis (PCA) of plasma analytes. Right: Comparison of PC1 and PC2 values. Significance determined with Mann–Whitney U test. *B*, Effect size and 95% CIs of plasma analytes significantly related to frailty in at least one of the 3 models, as determined by multiple least-squares linear regression analysis. *C*, Percent contribution (top) and variable coordinate (bottom) values for the top 20 contributors to PC1. Variables significantly associated with frailty in panel B are highlighted in purple. *D*, Levels of IL-6 and urokinase plasminogen activator surface receptor (uPAR) in PWH with frailty with Fried scores (FS) of 3 (n = 40) or 4+ (n = 19). Values from 2 participants with FS = 5 are shown in a darker purple color. Significance determined with Mann–Whitney U test.

To determine if the overall inflammatory milieu was different in PWH with and without frailty, we measured levels of 75 analytes in participant plasma (Supplementary Table 1) and used PCA to compare the overall analyte distribution in 2-dimensional space (Figure 1*A*). Our panel included cytokines, chemokines, growth factors, matrix metalloproteases, and other inflammatory components, only some of which have been evaluated in frail individuals. The groups were significantly different along principal component 1 (PC1), suggesting that a substantial proportion of the variability in systemic inflammatory analytes among PWH is explained by frailty status.

Using multiple least-squares linear regression analysis, we found that 19 analytes were significantly associated with frailty, 12 of which remained significant after adjusting for comorbidities (Figure 1*B*; Supplementary Table 2) and 10 of which were among the top 20 contributors to PC1 (Figure 1*C*). Despite the difference in smoking status between the donor groups (Table 1), incorporating smoking status in the model did not substantially affect our results, as 13 of the 19 analytes remained significantly associated with frailty after further adjustment for smoking status (Supplementary Figure 1 and Table 3). Levels of these analytes were highly correlated with each other (Supplementary Figure 2*A*), and all significant correlations were in a positive direction, although the relationships were less robust among PWH without frailty (Supplementary Figure 2*B*) than among PWH with frailty (Supplementary Figure 2*C*). Of the 19 analytes associated with frailty in at least one model, only IL-6 and urokinase plasminogen activator surface receptor (uPAR) levels were significantly elevated in those PWH with frailty who had 4 or more Fried frailty criteria (n = 20) than in those with 3 criteria (n = 40) (Figure 1*D*).

**Figure 2.**
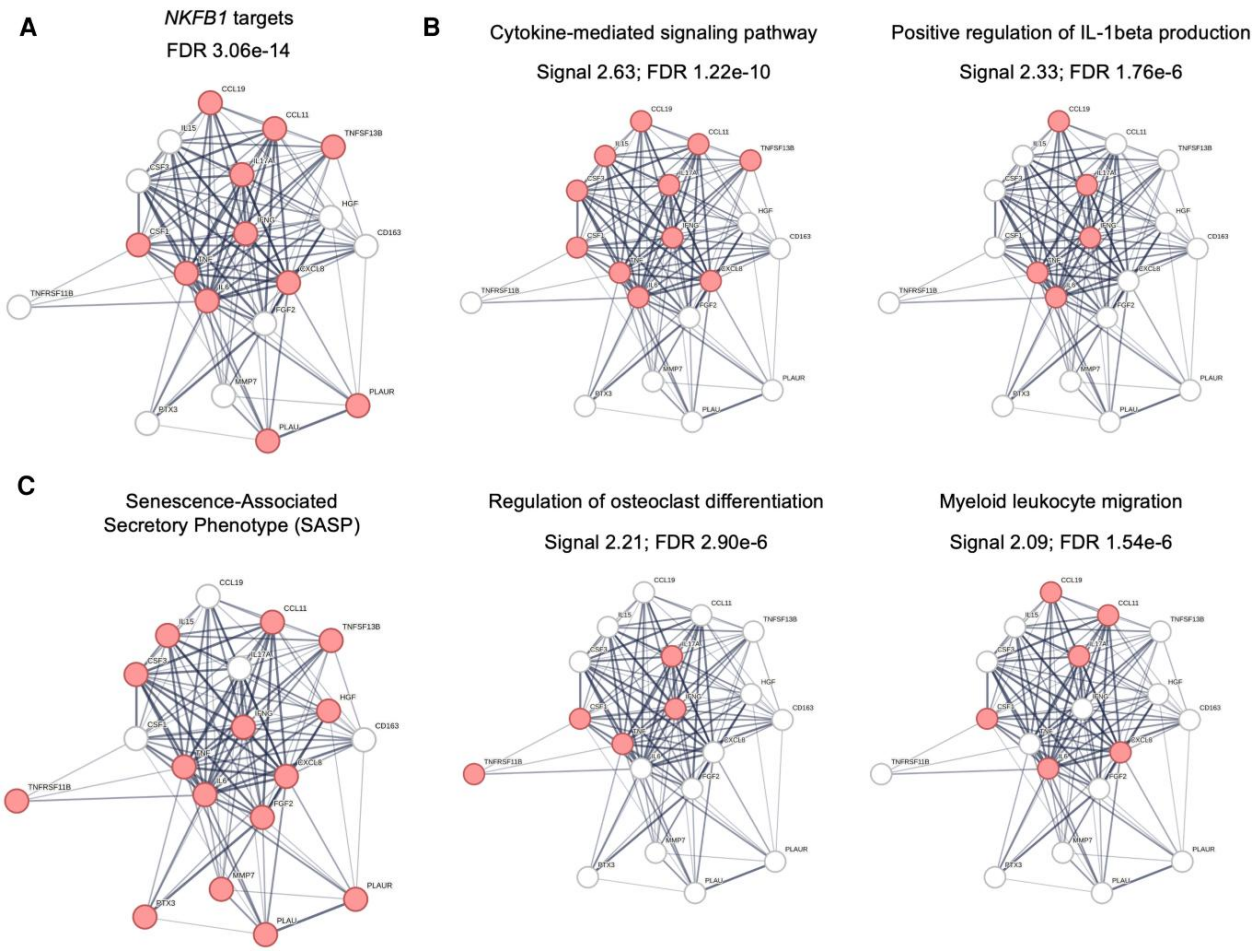
NF-κB-driven inflammation is associated with frailty in people with HIV. STRING database visualization of 19 frailty-associated proteins. *A*, Targets downstream of *NFKB1* were determined using the TRRUST database and are highlighted in red. *B*, Top 4 pathways of frailty-associated proteins; relevant nodes for each pathway are shown in red. *C*, Proteins associated with the senescence-associated secretory phenotype (SASP) are indicated in red. FDR, false discovery rate.

Notably, 11 of the 19 frailty-associated analytes were targets of the transcription factor nuclear factor kappa B (NF-κB) (Figure 2*A*), suggesting a link from innate inflammatory signals to frailty outcome, and pathway analysis revealed that the 4 most enriched signatures involved cytokine-mediated signaling, positive regulation of IL-1β production, regulation of osteoclast differentiation, and myeloid cell migration (Figure 2*B*). Our data are thus consistent with innate immune responses, inflammation, and signals of bone health being linked to frailty in PWH. Furthermore, IL-1β activity and NF-κB signaling are major drivers of the SASP [33], and 15 of the 19 frailty-associated factors have been linked to the SASP [22–30], of which 8 (53.3%) are also NF-κB targets (Figure 2*C*; Supplementary Table 4), suggesting that cellular senescence is a key pathogenic pathway in PWH with frailty.

**Figure 3.**
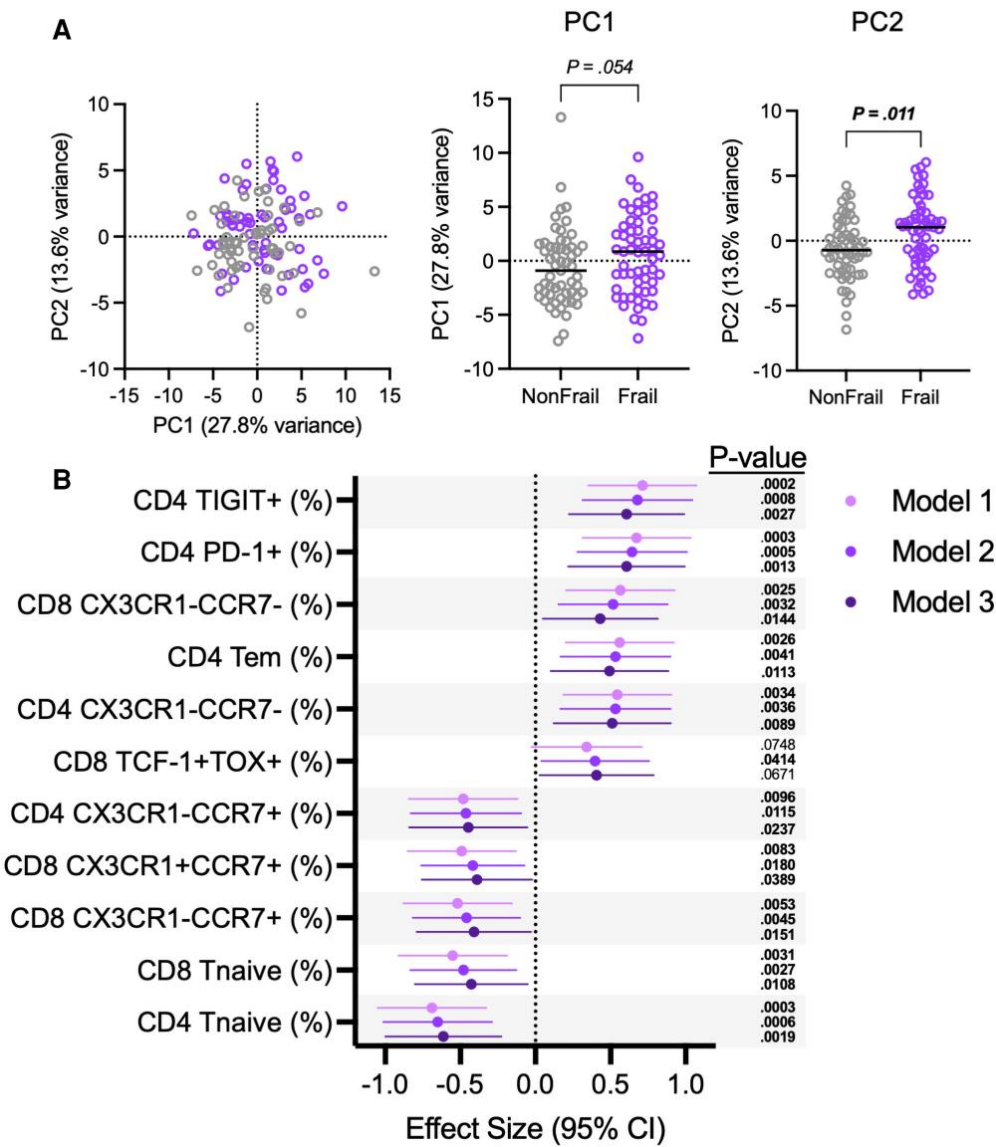
Association of T-cell phenotypes with frailty in people with HIV. Peripheral blood T-cell phenotypes were measured in people with HIV (PWH) without frailty (non-frail, n = 60) and in PWH with frailty (frail, n = 60). *A*, Left: Principal component analysis (PCA) of T-cell phenotypes. Right: Comparison of PC1 and PC2 values. Significance determined with Mann–Whitney U test. *B*, Effect size and 95% CIs of T-cell phenotypes significantly related to frailty in at least one of the 3 models, as determined by multiple least-squares linear regression analysis.

Next, we used flow cytometry to measure the expression of a number of phenotypic markers on peripheral blood CD4 and CD8 T cells in PWH with and without frailty (Supplementary Table 5), including markers of immune memory subpopulations (CD45RO and CCR7); markers of replicative senescence (CD57, CD28, intracellular p16^INK4a^); stemness (TCF-1); exhaustion and immune checkpoint molecules (TOX, PD-1, TIGIT); and the vascular tissue-homing receptor CX3CR1. Overall, T cells were substantially different between PWH with and without frailty (Figure 3*A*). We found that frailty was associated with 11 T-cell phenotypes in the fully adjusted model (Figure 3*B*; Supplementary Table 6). As with the soluble analytes, incorporating smoking status in the model did not substantially affect our results, as 10 of the 11 T-cell phenotypes remained significantly associated with frailty after further adjustment for smoking status (Supplementary Figure 3 and Table 7). As expected, these populations were highly correlated with each other (Supplementary Figure 4*A*) in both PWH without frailty (Supplementary Figure 4*B*) and in PWH with frailty (Supplementary Figure 4*C*), a result consistent with several of the phenotypes being reciprocal of each other (for negative relationships) and/or driven by similar factors (for positive ones). Interestingly, we did not detect differences in any of the 11 T-cell phenotypes between cells of PWH with 3 Fried frailty criteria and cells of PWH with 4 or more criteria (data not shown).

**Figure 4.**
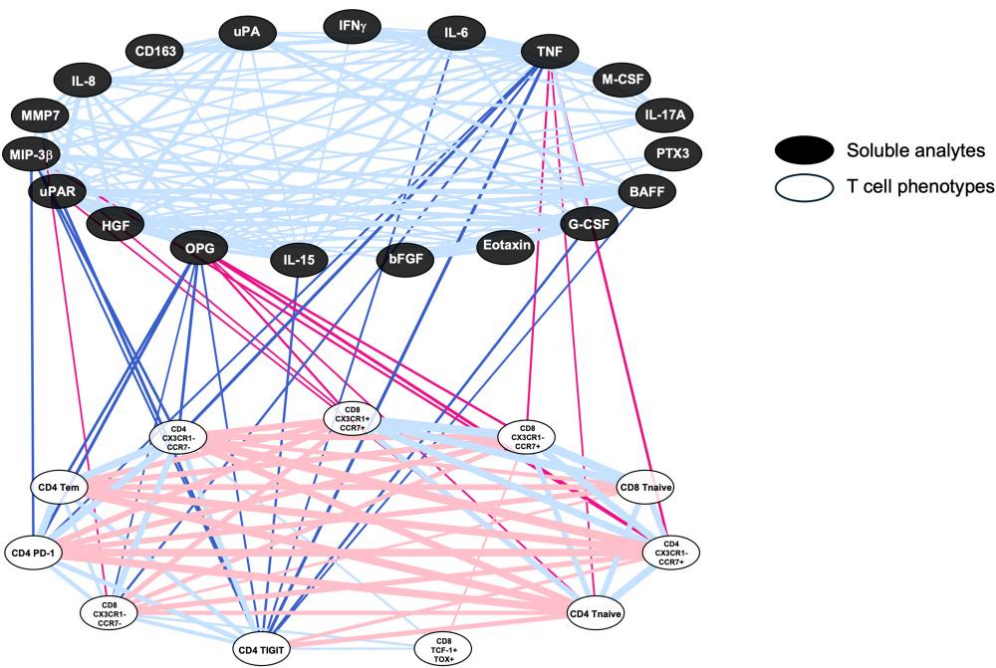
Correlation of frailty-associated soluble markers with frailty-associated T-cell phenotypes. The 19 soluble proteins associated with frailty (black nodes) were compared to the 11 frailty-associated T-cell phenotypes (white nodes) by Spearman correlation analysis among PWH with frailty (n = 59). Only significant correlations (*P ≤ .05*) are shown. Correlations with a negative rho value are in red, and correlations with a positive rho value are in blue. For emphasis, only correlations between soluble analytes and T-cell phenotypes are in bold colors.

We next investigated the relationships among the soluble analytes and T-cell phenotypes associated with frailty. Despite the robust interrelatedness of the soluble analytes (Supplementary Figure 2*C*) and the T-cell phenotypes (Supplementary Figure 4*C*) among PWH with frailty, we found that levels of only 8 of the soluble analytes were significantly correlated with T-cell phenotypes (Figure 4). The most striking of these were osteoprotegerin (OPG), TNF, and macrophage inflammatory protein-3 beta (MIP-3β), which were correlated with 9, 7, and 4 T-cell phenotypes, respectively. Among the T-cell phenotypes, CD4 T-cell expression of TIGIT was the most related to the soluble analytes, with 8 significant connections, including with OPG, TNF, and MIP-3β. Levels of OPG and TNF were also each correlated positively with expression of PD-1 and negatively with the naïve cell phenotype (Tnaive) on CD4 T cells. Taken together, our data demonstrate that innate inflammation, bone health, and the SASP are all associated with differentiation and exhaustion of CD4 T cells in PWH with frailty.

Lastly, we measured the ratio of CD4 to CD8 T cells, a surrogate marker for poor clinical outcomes in PWH and older PWoH [34, 35]. While the CD4/CD8 T cell ratio was not significantly related to frailty in any of our 4 models (Supplementary Tables 6 and 7), in an exploratory analysis, we found that the CD4/CD8 T-cell ratio was significantly negatively correlated with OPG levels in PWH with frailty (Supplementary Figure 5).

## DISCUSSION

In this study, we measured 75 soluble analytes in plasma and nearly 50 T-cell phenotypes from 120 PWH for their relationships among each other and to frailty, to clarify the immunological and inflammatory pathways that contribute to frailty outcomes in PWH. Most of the frailty-associated parameters remained associated after adjustment for potential confounders including age, sex, race/ethnicity, and comorbidities including hypertension, diabetes, CVD, and smoking. The soluble analytes were mainly driven by NF-κB signaling and were mostly components of the SASP, a heterogeneous constellation of cytokines and other factors produced and released by senescent cells that differ based on their cellular origin and on how senescence is induced in that cell [33]. While some of the factors, like IL-6 and TNF, have known associations with frailty in PWH [5], our work was has identified new relationships between soluble analytes and T-cell phenotypes, such as the links between OPG and PD-1 expression on CD4 T cells.

Interestingly, while we found many soluble SASP factors [22–29] were associated with frailty, the frailty-associated T-cell phenotypes were not those recognized as markers of senescence. For instance, neither intracellular expression of p16^INK4a^ nor surface expression of CD57—2 classic senescence markers in T cells [36, 37]—was associated with frailty. Whether this observation is due to the relatively young age of the cohort or unique to PWH is unclear and worth further study. We have previously identified a population of T cells that express CD57 and CX3CR1 and lack CD28 and CCR7 expression; we have termed this population “inflammescent” (Tinflamm) because of their robust inflammatory functionality (cytokine production, cytolytic capacity) and their expression of senescence markers (expression of CD57 and lack of CD28) [38]. While CD4 and CD8 Tinflamm were not associated with frailty, we did find that a greater proportion of CD4 and CD8 T cells had a CX3CR1-CCR7− phenotype among PWH with frailty in adjusted analyses. Among PWH with frailty, proportions of CD4 and CD8 T cells with the CX3CR1-CCR7− phenotype were significantly positively associated with the immune checkpoint markers PD-1 and TIGIT on CD4 T cells. Understanding if and how these T-cell populations may contribute to frailty outcomes will require future studies.

The proportions of naïve T cells were strongly negatively associated with frailty. These results are consistent with PWH with frailty having more memory T cells, perhaps indicative of more lifelong HIV antigen exposures or other chronic infections. Future work will need to address the antigen specificity of memory T cells in PWH with and without frailty, to determine if particular antigenic targets are driving memory T-cell expansion in individuals with frailty.

Besides the SASP, the major frailty-associated pathways were cytokine-mediated signaling, regulation of IL-1β production, and myeloid cell migration, with many of the proteins involved in more than one of these pathways. Intriguingly, we also found that signals of bone health (osteoclast differentiation) were related to frailty. Specifically, levels of OPG, a TNF receptor superfamily protein and SASP component [22], were significantly associated with frailty in all 4 models and were negatively correlated with the CD4/CD8 ratio among PWH with frailty. While not known to be directly regulated by NF-κB, OPG has been shown to promote NF-κB signals [39] and to inhibit osteoclast formation and prevent bone resorption by acting as a soluble decoy receptor for the receptor activator of NF-κB ligand (RANKL) [40]. Furthermore, other studies have linked OPG to frailty in PWoH [41] and found higher plasma OPG levels in PWH compared to levels in PWoH [42, 43]. We found that OPG was the analyte most strongly linked to T-cell phenotypes in PWH with frailty, with significant positive correlations with TIGIT and PD-1 expression, and the CX3CR1-CCR7− phenotype, on CD4 T cells, and significant negative correlations with the naïve CD4 T cells and CX3CR1-CCR7+ CD4 and CD8 T cells. But what is OPG doing in PWH with frailty—and is it a cause or consequence of immune activation? Importantly, activated T cells upregulate RANKL surface expression [44], and RANK expression on antigen presenting cells may be required for optimal memory T-cell responses upon antigen rechallenge [45]. Thus OPG, which can inhibit RANK/RANKL signals, might dampen T-cell function in PWH with frailty. Additional studies are needed to clarify whether the immune activities of OPG are related to osteoclast inhibition and how these disparate functions are linked to frailty in people with and without HIV.

Our study has some limitations. First, our objective was to examine inflammatory and immune associations with frailty among PWH, and so the relevancy of our findings to the general population is unclear. Second, our cohort of PWH with frailty was relatively young and mostly had a Fried score of 3, limiting our ability to observe differences based on more advanced frailty. Future work will need to revisit these findings in a cohort of individuals with more advanced age and/or worsened frailty measures. Third, our study was cross-sectional; whether any of these factors predict frailty development is unknown. As the HAILO cohort has plasma and cell samples stored over 7 years of observation, future research may be able to leverage these samples to address the predictive power of frailty-associated analytes and identify targets for interventions. Finally, low numbers of PWH with frailty but without low activity or weakness limited our power to assess differences based on individual frailty criteria. Strengths of our study include the well-characterized cohort of PWH who have been suppressed with ART for many years, the fairly large proportion of women included, and the extensive panels of inflammatory and T-cell biomarkers measured.

In conclusion, we have identified a cluster of NF-κB-driven soluble inflammatory SASP proteins associated with frailty in PWH. Of these, OPG and TNF were the most related to frailty-associated T-cell markers, such as CD4 T-cell expression of TIGIT and PD-1, and to low percentages of naïve CD4 and CD8 T cells. Our data are consistent with a pathogenic link between inflammation, T-cell activation, bone health, and frailty in PWH.

## Supplementary Material

jiag046_Supplementary_Data

## Supplementary Data

Supplementary materials are available at *The Journal of Infectious Diseases* online (http://jid.oxfordjournals.org/). Supplementary materials consist of data provided by the author that are published to benefit the reader. The posted materials are not copyedited. The contents of all supplementary data are the sole responsibility of the authors. Questions or messages regarding errors should be addressed to the author.
