# Supplementary material for: Frailty in People with HIV Is Linked to Inflammation, Bone Health, and T-Cell Exhaustion
Source: J Infect Dis. 2026 Feb 6;233(6):995–1004. doi: 10.1093/infdis/jiag046 (PMC13154846; doi:10.1093/infdis/jiag046)
Supplement: jiag046_Supplementary_Data [file jiag046_supplementary_data.zip › Figure S1_rev.pdf]

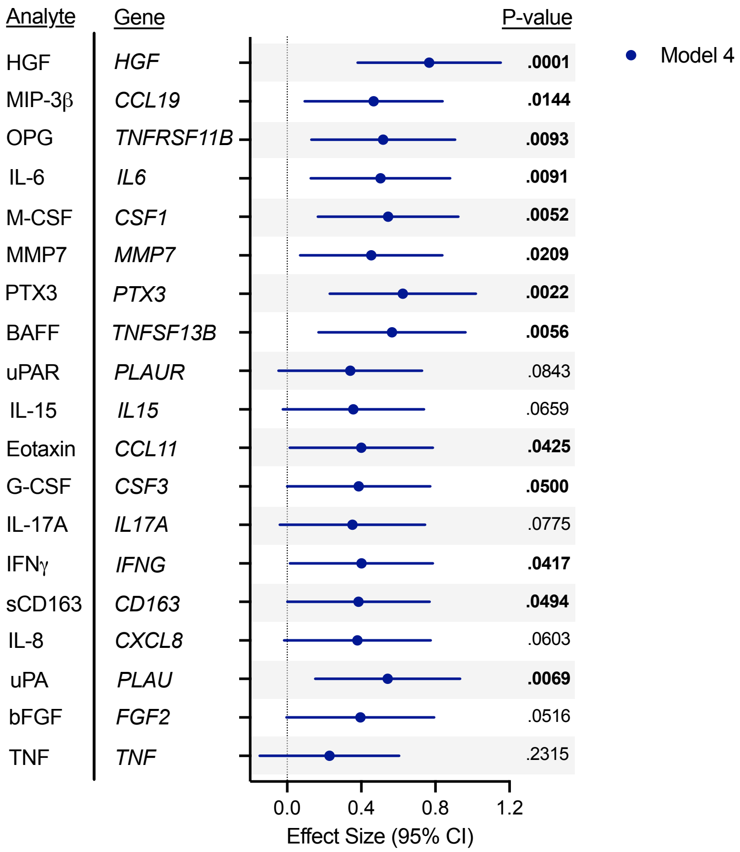

Figure S1. Association of soluble analytes with frailty in people with HIV. Soluble analytes in plasma were measured in people with HIV (PWH) without frailty (n=60) and in PWH with frailty (n=59). Effect size and 95% confidence intervals from Model 4 for the 19 plasma analytes significantly related to frailty in at least one of the first three models, as determined by multiple least-squares linear regression analysis.
