# Supplementary material for: Frailty in People with HIV Is Linked to Inflammation, Bone Health, and T-Cell Exhaustion
Source: J Infect Dis. 2026 Feb 6;233(6):995–1004. doi: 10.1093/infdis/jiag046 (PMC13154846; doi:10.1093/infdis/jiag046)
Supplement: jiag046_Supplementary_Data [file jiag046_supplementary_data.zip › Figure S2_rev.pdf]

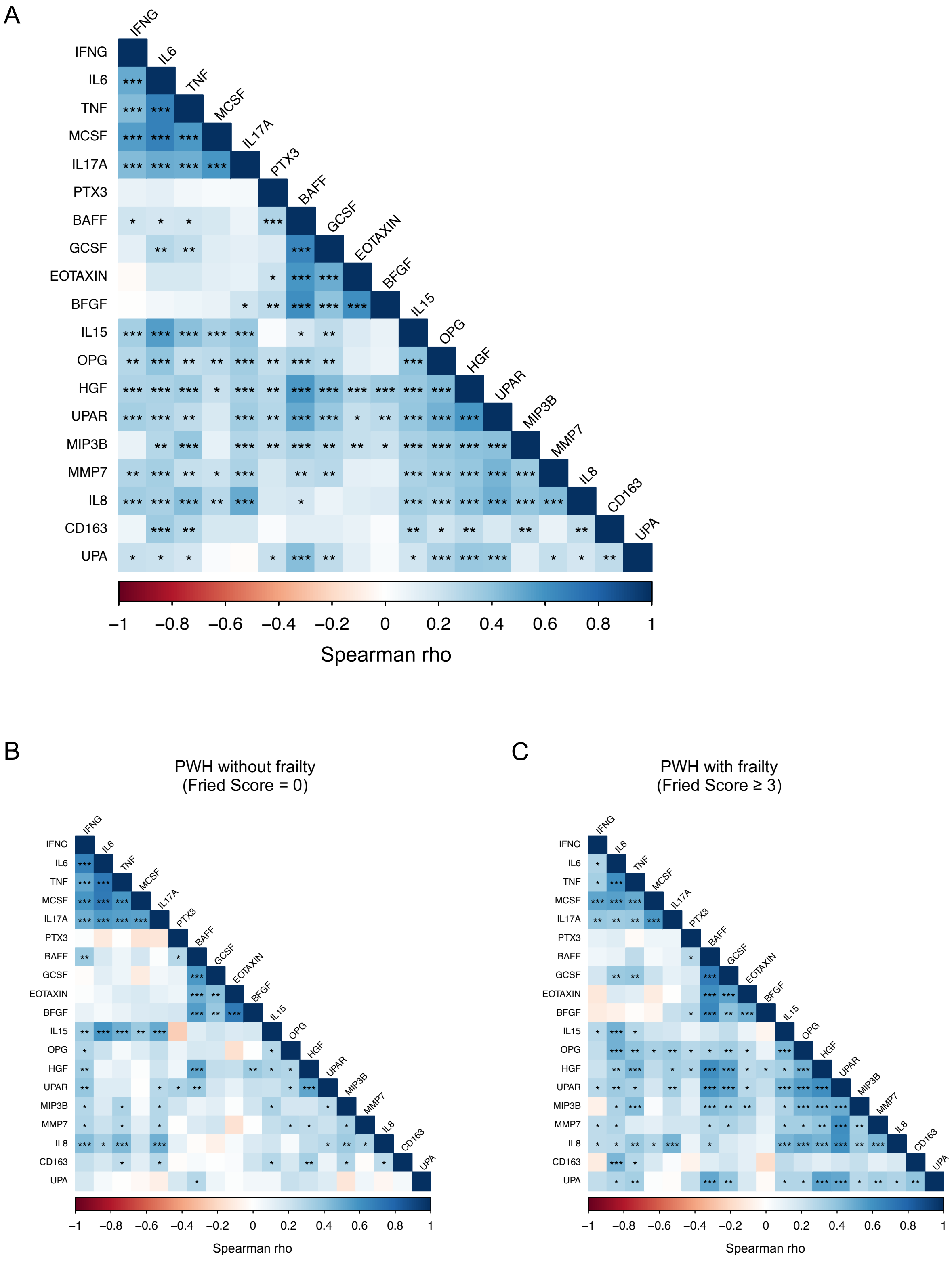

Figure S2. Correlations among frailty-associated soluble analytes. Spearman correlation analysis was performed on the 19 frailty-associated markers among **(A)** all participants (n=119); **(B)** among people with HIV (PWH) without frailty (n=60); and **(C)** among PWH with frailty (n=59). Colors indicate Spearman rho values. *$P<0.05$; **$P<0.01$; ***$P<0.001$.
