# Supplementary material for: Frailty in People with HIV Is Linked to Inflammation, Bone Health, and T-Cell Exhaustion
Source: J Infect Dis. 2026 Feb 6;233(6):995–1004. doi: 10.1093/infdis/jiag046 (PMC13154846; doi:10.1093/infdis/jiag046)
Supplement: jiag046_Supplementary_Data [file jiag046_supplementary_data.zip › Figure S3_rev.pdf]

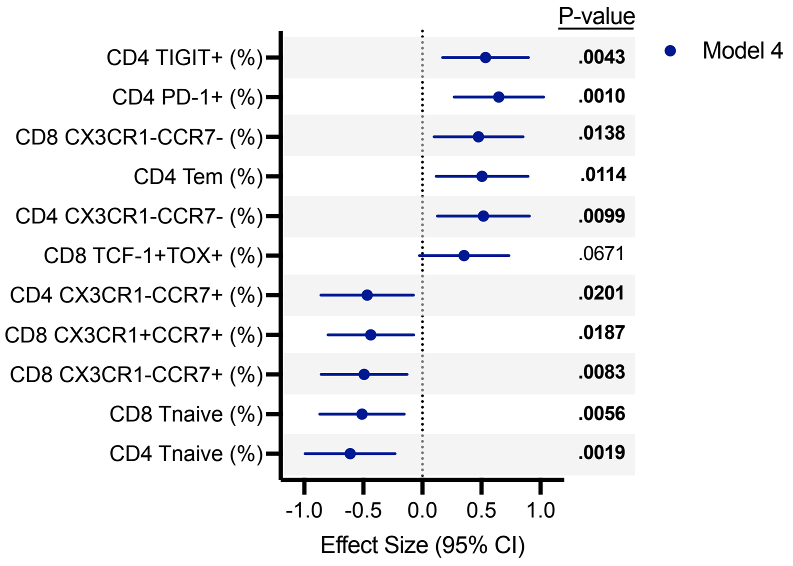

Figure S3. Association of T cell phenotypes with frailty in people with HIV. Peripheral blood T cell phenotypes were measured in people with HIV (PWH) without frailty (n=60) and in PWH with frailty (n=60). Effect size and 95% confidence intervals from Model 4 for the 11 T cell phenotypes significantly related to frailty in at least one of the first three models, as determined by multiple least-squares linear regression analysis.
