# Supplementary material for: Frailty in People with HIV Is Linked to Inflammation, Bone Health, and T-Cell Exhaustion
Source: J Infect Dis. 2026 Feb 6;233(6):995–1004. doi: 10.1093/infdis/jiag046 (PMC13154846; doi:10.1093/infdis/jiag046)
Supplement: jiag046_Supplementary_Data [file jiag046_supplementary_data.zip › Figure S5_rev.docx]

**Figure S5. Osteoprotegerin levels are negatively correlated with CD4/CD8 ratio.** Spearman correlation analysis of plasma osteoprotegerin (OPG) levels with CD4/CD8 T cell ratio in people with HIV with frailty (n=59), color coded by Fried score (FS).
