# Supplementary material for: Frailty in People with HIV Is Linked to Inflammation, Bone Health, and T-Cell Exhaustion
Source: J Infect Dis. 2026 Feb 6;233(6):995–1004. doi: 10.1093/infdis/jiag046 (PMC13154846; doi:10.1093/infdis/jiag046)
Supplement: jiag046_Supplementary_Data [file jiag046_supplementary_data.zip › INFDIS_jiag046_Supp Table Captions.docx]

**Table S1. Levels of Analytes in Plasma.**

Analytes were run in one of 3 Luminex panels. Data are presented as median and interquartile range (IQR).

**Table S2. Multivariable Linear Regression Models to Evaluate Soluble Frailty Associations.**

Effect size, 95% confidence intervals (95% CI), and P-values for the effect of frailty on plasma levels of each analyte as determined in 3 multivariable linear regression models. Model 1 was univariate. Model 2 was adjusted for age, sex, and race/ethnicity. Model 3 was adjusted for Model 2 parameters and for diabetes mellitus, hypertension, and cardiovascular disease.

**Table S3. Multivariable Linear Regression Model 4 to Evaluate Soluble Frailty Associations.**

Effect size, 95% confidence intervals (95% CI), and P-values for the effect of frailty on plasma levels of each analyte as determined in Model 4, adjusted for Model 3 parameters and smoking status.

**Table S4. NF-kB Targets and Senescence-Associated Secretory Phenotype Components among Frailty-associated Analytes.**

Characterization of frailty-associated analytes for whether they are a predicted target of NF-kB or a component of the senescence-associated secretory phenotype (SASP).

**Table S5. Peripheral Blood T Cell Phenotypes.**

Data are presented as median and interquartile range (IQR).

**Table S6. Multivariable Linear Regression Models to Evaluate T Cell Frailty Associations.**

Effect size, 95% confidence intervals (95% CI), and P-values for the effect of frailty on T cell phenotypes as determined in 3 multivariable linear regression models. Model 1 was univariate. Model 2 was adjusted for age, sex, and race/ethnicity. Model 3 was adjusted for Model 2 parameters and for diabetes mellitus, hypertension, and cardiovascular disease.

**Table S7. Multivariable Linear Regression Model 4 to Evaluate T Cell Frailty Associations.**

Effect size, 95% confidence intervals (95% CI), and P-values for the effect of frailty on plasma levels of each analyte as determined in Model 4, adjusted for Model 3 parameters and smoking status.
